# Data for optimizing Gamma Knife radiosurgery using the shot within shot technique

**DOI:** 10.1016/j.dib.2018.12.065

**Published:** 2018-12-23

**Authors:** Perry B. Johnson, Maria I. Monterroso, Fei Yang, Elizabeth Bossart, Amir Keyvanloo, Eric A. Mellon

**Affiliations:** University of Miami, Miller School of Medicine, Department of Radiation Oncology, United States

## Abstract

The tables included in this article will allow the user to implement shot within shot optimization for Gamma Knife radiosurgery planning and delivery. The method is intended to reduce treatment time when treating small to medium sized brain metastasis. The tables were previously developed by extracting profiles from Gamma Plan for three collimator settings and modeling their behavior when combined or prescribed at different isodose lines. For a given target size, the tables represent the optimal selection of shot weighting and prescription isodose line to reduce beam on time while maintaining an acceptable dose gradient. The method was recently validated in a large patient cohort and the data is this study is related to the research article titled “Clinical evaluation of shot within shot optimization for Gamma Knife radiosurgery planning and delivery” (Johnson et al., in press).

**Specifications table**

**Table:** 

Subject area	*Radiation Oncology*
More specific subject area	*Radiosurgery*
Type of data	*Tables*
How data was acquired	*Data was extracted from Leksell Gamma Plan v.10*
Data format	*Modeled data*
Experimental factors	*Data was processed using Matlab R2014B. The raw data was fit using a combination of Gaussian curves and modelled to create composite profiles.*
Experimental features	*Data interpolated for 4, 8, and 16 mm collimator settings available on the Elekta Gamma Knife Perfexion/Icon platforms*
Data source location	University of Miami, Miami, FL, 25.7883°N, 80.2162°W
Data accessibility	*Data is included within the article*
Related research article	*Johnson PB, Monterroso MI, Yang F, Bossart E, Keyvanloo A, Mellon EA. Clinical evaluation of shot within shot optimization for Gamma Knife radiosurgery planning and delivery. World Neurosurgery (in press)*[1]

**Value of the data**

• This data will allow clinicians to implement shot within shot optimization for Gamma Knife radiosurgery planning and delivery.
• The purpose of the technique is to reduce treatment times which can be lengthy and represent a burden to both the patient and staff.
• The optimization tables presented in the article pre-select treatment plans with prescription isodose lines greater than 50%.
• This represents a divergence from standard Gamma Knife practice and offers the potential to use this data to compare outcomes for patients treated using different methodologies and also modalities where higher prescription isodose lines are commonly utilized.

## 1 Data

The data presented in this article (Table 1, Table 2, Table 3, Table 4, Table 5, Table 6, Table 7) allow the user to implement shot within shot optimization for Gamma Knife (GK) radiosurgery planning and delivery of small to medium brain metastasis. The method requires a modern Gamma Knife platform, either the Icon or Perfexion system (Elekta Instruments, Stockholm Sweden). The user starts by creating a reference treatment plan prescribed at the 50% isodose line. The reference plan should also utilize the shot within shot technique whereby two shots are given the same stereotactic coordinates but different collimator settings. The weighting between the two shots is adjusted until the desired target coverage is achieved. The user then consults the optimization tables included herein and finds the row having the same collimator weighting for the reference plan as what was used to create the reference plan. The adjacent columns provide the details for the pre-selected optimized plan including the choice of prescription isodose line and collimator weighting. These settings should provide the same target coverage as the reference plan and have a gradient that is comparable to within 3%. Shot within shot optimization is not intended for use with very small or large lesions (< 4 mm or > 16 mm) or in cases where the lesion shape is very oblong or irregular.

**Table 1:** Optimization table used for the selection of the optimal IDL and collimator weighting based on a given reference plan. The data is read in rows whereby each reference plan corresponds to the adjacent optimized plan. The final column indicates the percentage of beam-on time saved when using the optimized plan.

**Reference plan**				**Optimized plan**				**Time reduction (%)**
**IDL (%)**	**4**	**8**	**16**	**IDL (%)**	**4**	**8**	**16**	
50	100	0	0	50	99	1	0	2.1
50	99	1	0	50	98	2	0	2.1
50	98	2	0	50	97	3	0	2.1
50	97	3	0	50	96	4	0	2.1
50	96	4	0	50	95	5	0	2.1
50	95	5	0	50	94	6	0	2.1
50	94	6	0	50	93	7	0	2.1
50	93	7	0	50	91	9	0	2.2
50	92	8	0	50	90	10	0	2.2
50	91	9	0	51	89	11	0	2.2
50	90	10	0	51	88	12	0	2.2
50	89	11	0	51	87	13	0	2.2
50	88	12	0	51	86	14	0	2.2
50	87	13	0	51	85	15	0	2.2
50	86	14	0	52	83	17	0	4.1
50	85	15	0	52	82	18	0	4.1
50	84	16	0	52	81	19	0	4.1
50	83	17	0	52	80	20	0	4.1
50	82	18	0	52	79	21	0	4.1
50	81	19	0	52	77	23	0	4.2
50	80	20	0	52	76	24	0	4.2
50	79	21	0	52	75	25	0	4.2
50	78	22	0	52	74	26	0	4.2
50	77	23	0	53	72	28	0	6.1
50	76	24	0	53	71	29	0	6.1
50	75	25	0	53	69	31	0	6.2
50	74	26	0	53	68	32	0	6.2
50	73	27	0	54	66	34	0	8.1
50	72	28	0	54	64	36	0	8.2
50	71	29	0	54	63	37	0	8.2
50	70	30	0	55	60	40	0	10.0

**Table 2:** Optimization table.

**Reference plan**				**Optimized plan**				**Time reduction (%)**
**IDL (%)**	**4**	**8**	**16**	**IDL (%)**	**4**	**8**	**16**	
50	69	31	0	55	59	41	0	10.0
50	68	32	0	56	57	43	0	11.7
50	67	33	0	57	54	46	0	13.4
50	66	34	0	58	51	49	0	15.1
50	65	35	0	60	46	54	0	18.3
50	64	36	0	62	41	59	0	21.2
50	63	37	0	63	38	62	0	22.6
50	62	38	0	64	35	65	0	24.0
50	61	39	0	69	25	75	0	30.1
50	60	40	0	74	14	86	0	35.5
50	59	41	0	81	0	100	0	41.8
50	58	42	0	80	1	99	0	40.9
50	57	43	0	80	0	100	0	40.9
50	56	44	0	79	0	100	0	40.1
50	55	45	0	79	0	100	0	40.1
50	54	46	0	78	0	99	1	39.3
50	53	47	0	78	0	99	1	39.2
50	52	48	0	77	0	99	1	38.4
50	51	49	0	76	0	99	1	37.5
50	50	50	0	76	0	99	1	37.4
50	49	51	0	75	0	99	1	36.5
50	48	52	0	74	0	99	1	35.6
50	47	53	0	74	0	99	1	35.6
50	46	54	0	73	0	99	1	34.6
50	45	55	0	72	0	98	2	33.7
50	44	56	0	72	0	98	2	33.6
50	43	57	0	71	0	98	2	32.6
50	42	58	0	71	0	98	2	32.6
50	41	59	0	70	0	98	2	31.5
50	40	60	0	70	0	98	2	31.5

**Table 3:** Optimization table.

**Reference plan**				**Optimized plan**				**Time reduction (%)**
**IDL (%)**	**4**	**8**	**16**	**IDL (%)**	**4**	**8**	**16**	
50	39	61	0	69	0	98	2	30.4
50	38	62	0	68	0	98	2	29.3
50	37	63	0	68	0	98	2	29.2
50	36	64	0	67	0	98	2	28.1
50	35	65	0	67	0	98	2	28.0
50	34	66	0	66	0	98	2	26.9
50	33	67	0	65	0	99	1	25.6
50	32	68	0	65	0	99	1	25.5
50	31	69	0	64	0	99	1	24.3
50	30	70	0	63	0	99	1	23.0
50	29	71	0	63	0	99	1	22.9
50	28	72	0	62	0	99	1	21.6
50	27	73	0	62	0	99	1	21.5
50	26	74	0	61	0	99	1	20.2
50	25	75	0	61	0	99	1	20.1
50	24	76	0	60	0	99	1	18.7
50	23	77	0	60	0	99	1	18.6
50	22	78	0	59	0	99	1	17.1
50	21	79	0	59	0	99	1	17.1
50	20	80	0	58	0	99	1	15.5
50	19	81	0	58	0	99	1	15.5
50	18	82	0	58	0	99	1	15.4
50	17	83	0	57	0	99	1	13.8
50	16	84	0	57	0	99	1	13.7
50	15	85	0	56	0	99	1	12.1
50	14	86	0	56	0	99	1	12.0
50	13	87	0	55	0	99	1	10.3
50	12	88	0	55	0	99	1	10.2
50	11	89	0	55	0	99	1	10.2
50	10	90	0	54	0	99	1	8.4

**Table 4:** Optimization table.

**Reference plan**				**Optimized plan**				**Time reduction (%)**
**IDL (%)**	**4**	**8**	**16**	**IDL (%)**	**4**	**8**	**16**	
50	9	91	0	54	0	99	1	8.3
50	8	92	0	53	0	99	1	6.5
50	7	93	0	53	0	99	1	6.4
50	6	94	0	53	0	99	1	6.3
50	5	95	0	52	0	99	1	4.4
50	4	96	0	52	0	99	1	4.3
50	3	97	0	52	0	99	1	4.2
50	2	98	0	51	0	99	1	2.3
50	1	99	0	51	0	99	1	2.2
50	0	100	0	0	0	0	0	0.0
50	0	99	1	0	0	0	0	0.0
50	0	98	2	0	0	0	0	0.0
50	0	97	3	0	0	0	0	0.0
50	0	96	4	0	0	0	0	0.0
50	0	95	5	0	0	0	0	0.0
50	0	94	6	0	0	0	0	0.0
50	0	93	7	0	0	0	0	0.0
50	0	92	8	0	0	0	0	0.0
50	0	91	9	0	0	0	0	0.0
50	0	90	10	0	0	0	0	0.0
50	0	89	11	0	0	0	0	0.0
50	0	88	12	0	0	0	0	0.0
50	0	87	13	0	0	0	0	0.0
50	0	86	14	0	0	0	0	0.0
50	0	85	15	0	0	0	0	0.0
50	0	84	16	0	0	0	0	0.0
50	0	83	17	51	0	81	19	2.2
50	0	82	18	51	0	80	20	2.2
50	0	81	19	51	0	79	21	2.2
50	0	80	20	51	0	78	22	2.2

**Table 5:** Optimization table.

**Reference plan**				**Optimized plan**				**Time reduction (% )**
**IDL (%)**	**4**	**8**	**16**	**IDL (%)**	**4**	**8**	**16**	
50	0	79	21	51	0	77	23	2.2
50	0	78	22	51	0	76	24	2.2
50	0	77	23	52	0	74	26	4.2
50	0	76	24	52	0	73	27	4.2
50	0	75	25	52	0	72	28	4.2
50	0	74	26	52	0	71	29	4.2
50	0	73	27	52	0	70	30	4.2
50	0	72	28	52	0	69	31	4.2
50	0	71	29	52	0	68	32	4.2
50	0	70	30	52	0	67	33	4.2
50	0	69	31	52	0	66	34	4.2
50	0	68	32	54	0	62	38	8.0
50	0	67	33	54	0	61	39	8.0
50	0	66	34	54	0	60	40	8.0
50	0	65	35	54	0	59	41	8.0
50	0	64	36	54	0	58	42	8.0
50	0	63	37	56	0	54	46	11.6
50	0	62	38	56	0	53	47	11.6
50	0	61	39	56	0	52	48	11.6
50	0	60	40	58	0	48	52	14.9
50	0	59	41	58	0	47	53	14.9
50	0	58	42	59	0	44	56	16.5
50	0	57	43	61	0	40	60	19.5
50	0	56	44	61	0	39	61	19.5
50	0	55	45	62	0	36	64	20.9
50	0	54	46	64	0	32	68	23.6
50	0	53	47	65	0	29	71	25.0
50	0	52	48	66	0	27	73	26.2
50	0	51	49	68	0	22	78	28.6
50	0	50	50	69	0	19	81	29.8

**Table 6:** Optimization table.

**Reference plan**				**Optimized plan**				**Time reduction (%)**
**IDL (%)**	**4**	**8**	**16**	**IDL (%)**	**4**	**8**	**16**	
50	0	49	51	71	0	15	85	32.0
50	0	48	52	72	0	12	88	33.1
50	0	47	53	75	0	6	94	36.1
50	0	46	54	77	0	1	99	38.0
50	0	45	55	76	0	1	99	37.1
50	0	44	56	76	0	0	100	37.1
50	0	43	57	75	0	0	100	36.2
50	0	42	58	74	0	0	100	35.3
50	0	41	59	73	0	1	99	34.2
50	0	40	60	72	0	1	99	33.3
50	0	39	61	72	0	0	100	33.3
50	0	38	62	71	0	0	100	32.2
50	0	37	63	70	0	0	100	31.2
50	0	36	64	69	0	1	99	30.1
50	0	35	65	69	0	0	100	30.1
50	0	34	66	68	0	0	100	29.0
50	0	33	67	67	0	1	99	27.8
50	0	32	68	67	0	0	100	27.7
50	0	31	69	66	0	0	100	26.6
50	0	30	70	65	0	1	99	25.3
50	0	29	71	65	0	0	100	25.3
50	0	28	72	64	0	0	100	24.1
50	0	27	73	63	0	1	99	22.7
50	0	26	74	63	0	0	100	22.7
50	0	25	75	62	0	0	100	21.4
50	0	24	76	61	0	1	99	19.9
50	0	23	77	61	0	0	100	19.9
50	0	22	78	60	0	1	99	18.4
50	0	21	79	60	0	0	100	18.4
50	0	20	80	59	0	1	99	16.9

**Table 7:** Optimization table.

**Reference plan**				**Optimized plan**				**Time reduction (%)**
**IDL (%)**	**4**	**8**	**16**	**IDL (%)**	**4**	**8**	**16**	
50	0	19	81	59	0	0	100	16.9
50	0	18	82	58	0	0	100	15.3
50	0	17	83	58	0	0	100	15.3
50	0	16	84	57	0	0	100	13.7
50	0	15	85	57	0	0	100	13.6
50	0	14	86	56	0	0	100	12.0
50	0	13	87	56	0	0	100	11.9
50	0	12	88	55	0	0	100	10.2
50	0	11	89	55	0	0	100	10.1
50	0	10	90	54	0	1	99	8.2
50	0	9	91	54	0	0	100	8.2
50	0	8	92	53	0	1	99	6.3
50	0	7	93	53	0	0	100	6.3
50	0	6	94	52	0	1	99	4.3
50	0	5	95	52	0	0	100	4.3
50	0	4	96	51	0	1	99	2.3
50	0	3	97	51	0	0	100	2.3
50	0	2	98	51	0	0	100	2.2
50	0	1	99	0	0	0	0	0.0
50	0	0	100	0	0	0	0	0.0

## 2 Experimental design, materials, and methods

A full description of the methods used to create the optimization tables included in this work can be found in Johnson et al. [2]. The implementation of the technique is described further in Johnson et al. [1]. In short, profiles were extracted from Leksell Gamma Plan v.10 for each of the three collimator settings available on the GK Perfexion (4, 8, and 16 mm). The profiles were fit using a combination of Gaussian curves and parameterized using Matalb R2014b (MathWorks, Natick, MA). The parameterized data allowed for the recreation of beam profiles for shot within shot plans having prescription isodose lines ranging from 40% to 90% and a various collimator ratios when using the shot within shot technique. An optimization algorithm was written to compare “reference” plans prescribed at the 50% isodose line with other suitable options. These “optimized plans” are prescribed at higher isodose lines and have a different collimator weighting than the reference plan. These two parameters both affect target coverage and the dose gradient and thus can be adjusted accordingly to maintain the same target coverage, conformity, and dose gradient as the reference plan. The optimized plan having the minimum beam-on time was selected for each reference plan. The details for these plans are included within this work such that other users can recreate them for their own clinical cases once they have created a reference plan specific to a given target (see Table 1, Table 2, Table 3, Table 4, Table 5, Table 6, Table 7).

## References

[1] Johnson P.B., Monterroso M.I., Yang F., Bossart E., Keyvanloo A., Mellon E.A. (2019). Clinical evaluation of shot within shot optimization for Gamma Knife radiosurgery planning and delivery. World Neurosurg..

[2] Johnson P.B., Monterroso M.I., Yang F., Mellon E.A. (2017). Optimization of the prescription isodose line for Gamma Knife radiosurgery using the shot within shot technique. Radiat. Oncol..

