# Supplementary material for: Data for optimizing Gamma Knife radiosurgery using the shot within shot technique
Source: Data Brief. 2018 Dec 23;22:620–6. doi: 10.1016/j.dib.2018.12.065 (PMC6327102; doi:10.1016/j.dib.2018.12.065)
Supplement: Supplementary file 1 — Supplementary material [file mmc1.docx]

**Disclosures**

The authors did not receive specific funding for this study. All data collection and analysis was performed as part of the routine clinical practice at the academic facility. The authors have no competing interest to disclose.
